# Management of Precancerous Lesions of the Uterine Cervix according to Demographic Data

**DOI:** 10.5402/2011/301680

**Published:** 2010-10-27

**Authors:** Olga Modinou, Lykourgos Liaropoulos, Dafni Kaitelidou, kyriakos Kioulafas, Eleni-Maria Theodoraki

**Affiliations:** ^1^Department of Nursing, Centre for Health Services Management and Evaluation, National and Kapodistrian University of Athens, Dilou 1A, Goudi, 115 27 Athens, Greece; ^2^Department of Economics, National and Kapodistrian University of Athens, Pesmazoglou 8, 105 59 Athens, Greece; ^3^Department of Statistics and Actuarial-Financial Mathematics, University of the Aegean, Karlovassi, 83200 Samos Island, Greece

## Abstract

*Aims*. Worldwide, cervical cancer is the fifth most deadly cancer in women, but screening prevents cancer by detecting precancerous lesions. The purpose of this study is to present the treatment profile for precancerous lesions of the uterine cervix, according to demographic data. *Methods*. An annual retrospective study was conducted in two public primary health care centres in Greece. The total number of Pap smears and colposcopies performed as well as the management of women with cervical intraepithelial neoplasia was collected and analysed. *Results*. Demographic characteristics and correlations with levels of Cervical Intraepithelial Neoplasia (CIN) and treatment path are presented. For each case, we noted the patients' age, the marital and educational status, and the professional and insurance type. From a total of 238 diagnostic procedures, 118 (49.5%) showed precancerous lesions, 83.3% of these were high grade while 16.7% were low grade. *Conclusions*. This study provides an estimate of the extensiveness of precancerous lesions of the uterine cervix. Management of CIN should be accounted for when balancing the benefits and unfavourable effects of this screening.

## 1. Introduction

Cervical cancer remains the second most common cancer in women worldwide and the first in most developing countries, where 80% of cases occur. Cervical cancer is developed through precancerous stages, termed cervical intraepithelial neoplasia CIN, and caused by infection with human papillomavirus (HPV) [1]. 

Epidemiological studies have clearly demonstrated that in countries that achieve frequent screening, the incidence and the mortality rates of cervical cancer have decreased considerably over the last four decades [2–4]. Screening with the use of PAP test prevents cancer by detecting precancerous lesions (CIN), and early treatment of precancerous lesions can prevent progression to cancer [5, 6]. Conventional cytology allows the detection of abnormal findings leading to a colposcopy with biopsy for a definitive diagnosis if there is persistence of atypical squamous cells with undetermined significance or low-grade or high-grade squamous intraepithelial lesion (LSIL, HSIL). Generally, ASCUS corresponds to ill-defined abnormalities of superficial cells and represents 2% to 3% of all cervical smears. Additionally, biopsy shows CIN grades II-III in 5% to 10% of ASCUS cases. LSIL corresponds to mildly abnormal squamous cells and is found in 1% to 2% of all cervical smears. In most cases it regresses spontaneously, especially in young patients. HSIL corresponds moderately to severely abnormal squamous cells and represents 0.5% of all cervical smears [7]. 

Management options for LSIL (CIN1 grade) on histology vary, ranging from simple observation to excisional therapies. Patients with persistent LSIL should be treated chiefly with the use of office-based ablative therapies. Management guidelines for HSIL (CIN2/3 grade) are well established and recommend colposcopy-directed biopsy. Cold-knife conization or electroconization should be performed in all patients with biopsy-confirmed HSIL in order to exclude invasive disease [1].

The annual incidence for CIN 1 and CIN 2, 3 in the US is 1.6 and 1.2 per 1,000 women, respectively. The incidence is highest among women aged from 21 to 30 years (3.3 and 3.6 per 1,000) and women aged from 31 to 40 years (2.9 and 2.7 per 1,000) [8]. In Spain, it is estimated that 7.6 million Pap smears are performed annually, with 3.5% of routine Pap smears to be abnormal and with 40,530 women to be annually diagnosed with CIN 1, 26,243 with CIN 2, and 28,423 with CIN 3 [9]. In France, Estimated 6,111,787 Pap smears were performed in 2004, including 222,350 abnormal (3.9%) and 63,616 followup smears. In total, 58,920 cervical biopsies and 52,525 HPV tests were performed after an abnormal Pap smear [10]. Estimated 6.4 million women aged between 25 and 69 years undergo screening annually in Italy (1.2 million and 5.2 million through organized and opportunistic screening programs, resp.). Approximately 2.4% of tests have positive findings, and there are approximately 21,000 cases of CIN1 and 7,000–17,000 cases of CIN2/3 [11]. In Greece, cervical scrapes from 841 women in 2006, which were obtained for cytological evaluation, showed that the normal Pap test results or benign cellular changes were 45,8% of the women, atypical squamous cells of undeterminated significance (ASCUS) were 23,2%, low-grade squamous intraepithelial lesion (LSIL) was 27,9%, and high-grade squamous intraepithelial lesion (HSIL) was 3,1% [12].

This study aims to present the management of precancerous lesions of the uterine cervix, according to cytological and epidemiological aspects as well as the treatment methods adopted.

## 2. Methods

The study population consisted of 238 women screened for a regular gynaecological control in the gynaecological outpatient clinic of two public primary health care centres in Greece (a general one where all patients are admitted and a hospital specialized on cancer cases). Registry was annual and lasted from January 2007 to January 2008. Possible factors associated with CIN were examined like women age, marital and educational status, and professional and insurance type. Other characteristics like smoking, HPV, the season of women's visit at the hospital were also considered. Statistical tests used for comparisons of factors were Fisher exact test and Pearson Chi-square in cases where the latter was not possible to be implemented. Twoindependent samples *t*-test and one-way ANOVA were applied for comparisons of continuous variables between groups. Quantitative variables are expressed as Mean  ±  SD, and *P*-values smaller than  .05 were considered statistically significant. Analysis was performed with the statistical software SPSS 17.

## 3. Results

**Figure 1:**
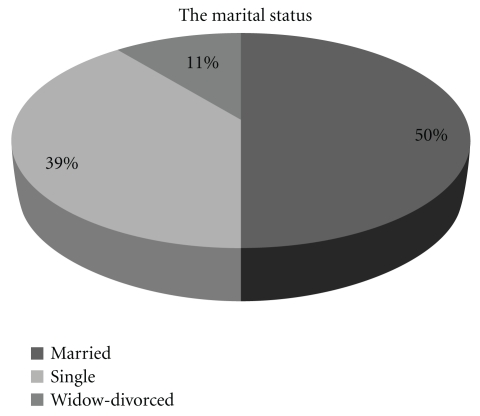
The marital status of the study population.

**Figure 2:**
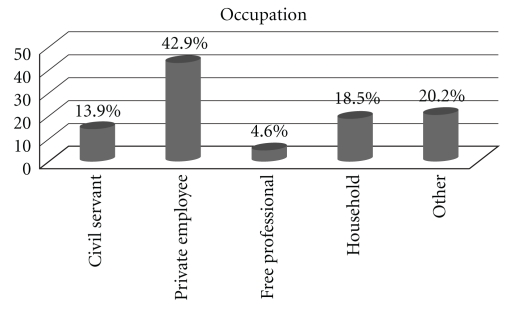
The occupation of the study population.

Population age was 37.03  ±  10.47 years (Mean  ±  SD). Almost half of women (49.6%) had at least one child, and most of them had two children. As far as education is concerned, 55.9% completed high school, 42% higher education while the remaining 2.1% elementary. The marital status and occupation of women who participated in this study are presented in Figures 1 and 2, respectively.

Demographic features according to the stages of dysplasia and the type of medical treatment are described in Tables 1, 2, and 3. As shown, the majority of women were married, young (17–30 years old), and private employees, who had completed high school. The marital status of patients seems to affect the dysplasia grade (*P* = .02). Mostly unmarried women were diagnosed with CIN1 (49.0%), divorced women with CIN2 (12.2%), and married women and widows with the high-grade intraepithelial lesion CIN3, with rates 67.9% and 7.1%, respectively. A positive relation was observed when we compared the education status (*P* = .64), the occupation (*P* = .66), and the insurance (*P* = .99) with the grade of dysplasia of the studied woman, without reaching statistical significance. The majority of the women with higher education (57,7%) and public insurance (50.8%), occupied as private employees (44,2%), were diagnosed with higher grades of dysplasia, CIN3, and ASC/ASCUS. 

Moreover, there was a significant variation of the type of abnormalities according to the age group. Specifically, young women (17–30 years old) did not have hgsil, but atypical squamous cells (ASCs) or atypical squamous cells of undetermined significance (ASCUS) (35%). Older women (30–35 years) were detected with CIN1 and at older ages (36–50 years) with CIN3. Specifically there was a rate of 17.9% of 36–40 years old women with CIN detected, a rate of 25% at women of 41–45 years, and a rate of 28.6% at women of 46–50 years old. Middle aged ones (51–55 years old) had a reduced level of cervical intraepithelial neoplasia CIN2 (12.2%), and women over 55 years old did not have atypical squamous cells (ASCs) or atypical squamous cells ASC of undetermined significance (ASCUS) dysplasia (ASC/ASCUS) (4.2%). It should be mentioned that patients with ASC/ASC-US had HPV infection rate of 47 5%, patients with cervical intraepithelial neoplasia CIN1 had 49%, and finally patients with CIN2 and CIN3 had 48.8% and 42.9%, respectively, (data not shown).

Additionally, several other factors were considered in order to study possible correlations according to CIN. For that purpose, smoking status was examined, but with no statistically significant results. Twenty-two point one percent of women smokers developed cervical intraepithelial neoplasia CIN1, 17.1% CIN2, and CIN3 10.7%, compared to nonsmokers, while the number of daily consumption of cigarettes did not appear to be associated with dysplasia (data not shown).

When the same analysis was performed for women with confirmed cancer, the majority of married women were treated with surgical procedures, such as LLETZ (83,3%), loop electrosurgical excisional procedure (54,8%), and diathermic conization electrosurgical excisional procedure (70.5%). A statistically significant correlation was established (*P* < .001) when we compared the management of the precancerous and cancerous lesions with marital status. A positive relation was observed when we compared the education status (*P* = .75), the occupation (*P* = .26), and the insurance (*P* = .07) with the management of cancerous lesions of the studied woman, without reaching statistical significance.

## 4. Discussion

The development and implementation of population control can lead to prevention and early detection of precancerous lesions of the cervix. Cytological screening is the basic method in the early finding and screening of precancer and cancer of the cervix. The colposcopical method is widely used in the every-day practice and is mostly used after obtaining the abnormal Pap smear test or after positive result from HPV test. All these diagnostic procedures have to combine together with the purpose of better early diagnosis and adequate treatment of these disease. 

Regarding the treatment method followed, we observed that the greater the degree of dysplasia of the cervix was, the higher the degree of treatment was, ranging from conservative management to surgical excision treatment. The largest proportion of patients treated conservatively with 6-month and annual retesting with Pap test and colposcopy was with simple informal ASC-ASCUS (89.17%). For CIN1, the percentages of patients treated conservatively with surgical resection of the cervix were at the same level, 46.94% and 53.07%, while for HGSIL (CIN2/3), the main treatment was surgical reconstruction with conical resection of the cervix. According to the guidelines referred to in other studies, the management of high-grade intraepithelial lesions (CIN2/3) is colposcopy, biopsy with or without intracervical curettage, and surgical treatment with cervical cone excision [13]. According to the marital status of our study population, dysplasia grade seems to be differentiated especially in married women, with the rate of 67.9% for the high-grade intraepithelial lesion. The risk of high-grade intraepithelial lesion is almost 2 times greater in married women than in single ones, who happen to be in higher median age, and the risk increases with time since the last normal smear or with lower frequency of screening. A study in China showed that, in Beijing, married women at the age of 30–34 are the high-risk group in CIN incidence compared to women in other marital status [14].

The association between smoking status and the degree of dysplasia was not confirmed, in contrary to other studies, where smoking intensity is an independent risk factor for high-grade CIN in young women, after controlling the cervical HPV infection. Other papers also indicate that current smokers are as likely to be diagnosed twice with high-grade CIN as nonsmokers [15] and also that current cigarette smoking is a risk factor, closely interrelated in that the high-risk HPV types are significantly more frequent in current smokers than in nonsmokers [16]. Also, despite the fact that it cannot be concluded that smoking is a genuine cause of cervical neoplasia, results from other studies support the hypothesis that smoking is a true risk factor in cervical neoplasia [17–19]. 

Our study included women with positive test and cervical intraepithelial abnormalities, so results cannot be extrapolated to the total female population in Greece. However, since the outpatient clinics are referral ones, the results give us important information on the cytological and epidemiological aspects of precancerous lesions of the uterine cervix of Greek women and the therapy provided according to current medical practice. In addition, precancerous lesions of the cervix are treatable with minimally invasive surgical methods, so women in early stages of precancerous lesions or patients undergoing such treatment should be rechecked at regular intervals in order to prevent the recurrence or progression of the disease. Larger epidemiological studies in different regions of the country are needed in order to report on the epidemiology of precancerous lesions of the uterine cervix in Greece.

## 5. Conclusion

In the absence of a national screening programme, the present clinical study is an effort of utmost importance for the early detection and prevention of cervical cancer. Additional effort is needed from the Greek Government to improve the information provided and the education of women on the prevention of cervical cancer and also to develop an organized national policy, by call and recall of all women for the regular diagnostic procedures.

## Figures and Tables

**Table 1:** Patient demographics by hospital.

Characteristics		Hospital	
		General hospital (%)	Cancer hospital (%)
Marital status	Single	49.5	50.3
	Married	39.5	38.8
	Widow-divorced	11.0	10.9

Education	Illiterate	0.0	0.0
	Elementary primary school	3.7	0.8
	High school	63.3	49.6
	University	33.0	49.6

Occupation	Civil servant	12.8	14.7
	Private employee	46.8	39.5
	Free professional	4.6	4.7
	household	21.1	16.3
	Other	14.7	24.8

Insurance fund	Public sector	17.4	18.6
	Social security institute	51.4	47.3
	Insurance fund for craftsmen and small traders	13,8	15,6
	Lawyers insurance fund	4.6	4.7
	Mariners' pension fund	6.4	8.5
	Agricultural insurance organization	6.4	5.4

**Table 2:** Patient demographics according to dysplasia grade.

Characteristics	CIN grade								*P*-value*
	CIN1		CIN2		CIN3		ASC/ASCUS		
	*N*	%	*N*	%	*N*	%	*N*	%	
Marital status									.02*
Married	19	38.8	23	56.1	19	67.9	58	48.3	
Single	24	49.0	11	26.8	5	17.9	53	44.2	
Widow-divorced	6	12.2	7	17.1	4	14.2	9	7.5	

Education									.64*
Illiterate	0	0.0	0	0.0	0	0.0	0	0.0	
Elementary primary school	0	0.0	0	0.0	1	3.6	4	3.3	
High school	25	51.0	22	53.7	17	60.7	69	57.5	
University	24	49.0	19	46.3	10	35.7	47	39.2	

Occupation									.66**
Civil servant	9	18.4	6	14.6	3	10.7	15	12.5	
Private employee	20	40.8	18	43.9	11	39.3	53	44.2	
Free professional	3	6.1	2	4.9	0	0.0	6	5.0	
Household	4	8.2	9	22.0	6	21.4	25	20.8	
Other	13	26.5	6	14.6	8	28.6	21	17.5	

Insurance Fund									.99*
Public sector	10	20.4	9	22.0	5	17.9	19	15.8	
Social security institute	24	49.0	18	43.9	14	50.0	61	50.8	
Insurance fund for craftsmen and small traders	7	14,3	5	12,3	3	10.7	20	16.7	
Lawyers insurance fund	3	6.1	2	4.9	1	3.6	5	4.2	
Mariners' pension fund	3	6.1	4	9.8	2	7.1	9	7.5	
(Hellenic) agricultural insurance organization	2	4.1	3	7.3	3	10.7	6	5.0	

Age (mean ± SD)	49	35 ± 11	41	39 ± 10	28	43 ± 8	120	35 ± 11	<.001***

*Fisher's exact test

**Pearson Chi-square

***One-way ANOVA.

**Table 3:** Demographics of patients by treatment path.

	Conservative treatment		Cryotherapy		Diathermic conization electrosurgical excisional procedure		Loop electrosurgical excisional procedure		LLETZ		*P*-value
	*N*	%	*N*	%	*N*	%	*N*	%	*N*	%	
Marital status											<.001*
Married	55	41.4	5	38.5	31	70.5	23	54.8	5	83.3	
Single	64	48.1	7	53.8	7	15.9	14	33.3	1	16.7	
Widow-divorced	14	10.5	1	7.7	6	13.6	5	11.9	0	0.0	

Education											.75*
Illiterate	0	0.0	0	0.0	0	0.0	0	0.0	0	0.0	
Elementary primary school	3	2.3	0	0.0	1	2.3	1	2.4	0	0.0	
High school	81	60.9	6	46.2	21	47.7	22	52.4	3	50.0	
University	49	36.8	7	53.8	22	50.0	19	45.2	3	50.0	

Occupation											.26**
Civil servant	15	11.3	2	15.4	8	18.2	8	19.0	0	0.0	
Private employee	54	40.6	9	69.2	18	40.9	20	47.6	1	16.7	
Free professional	8	6.0	0	0.0	3	6.8	0	0.0	0	0.0	
Household	24	18.0	2	15.4	9	20.5	6	14.3	3	50.0	
Other	32	24.1	0	0.0	6	13.6	8	19.0	2	33.3	

Insurance Fund											.07**
Public sector	19	14.3	2	15.4	11	25.0	11	26.2	0	0.0	
Social security institute	63	47.4	10	76.9	19	43.2	23	54.8	2	33.3	
Insurance fund for craftsmen and small traders	23	17.3	0	0.0	8	18,2	3	7.1	1	16.7	
Lawyers insurance fund	8	6.0	1	7.7	1	2.3	0	0.0	1	16.7	
Mariners' pension fund	12	9.0	0	0.0	4	9.1	2	4.8	0	0.0	
(Hellenic) agricultural insurance organization	8	6.0	0	0.0	1	2.3	3	7.1	2	33.3	

Age (mean ± SD)	133	35 ± 11	13	34 ± 6	44	41 ± 10	42	39 ± 10	6	43 ± 10	<.001***

*Fisher's exact test

**Pearson Chi-square

***One-way ANOVA.
